# Relationship of mineral elements in sheep grazing in the highland agro-ecosystem

**DOI:** 10.5713/ajas.18.0955

**Published:** 2019-04-15

**Authors:** Qingshan Fan, Zhaofeng Wang, Shenghua Chang, Zechen Peng, Metha Wanapat, Saman Bowatte, Fujiang Hou

**Affiliations:** 1 State Key Laboratory of Grassland Agro-ecosystems; Key Laboratory of Grassland Livestock Industry Innovation, Ministry of Agriculture; College of Pastoral Agriculture Science and Technology, Lanzhou University, Lanzhou 730020, China; 2 Tropical Feed Resources Research and Development Center (TROFREC), Department of Animal Science, Faculty of Agriculture, Khon Kaen University, Khon Kaen 40002, Thailand

**Keywords:** Macro and Micro Mineral, Deficiency, Soil-plant-animal, Qilian Mountain Grassland, Correlations

## Abstract

**Objective:**

Minerals are one of the important nutrients for supporting the growth of sheep grazing in the highland, northwest of China. The experiment was conducted to investigate the relationship of both macro and micro minerals in sheep grazing in the highlands of six districts located in the Qilian Mountain of China.

**Methods:**

Samples of herbage (n = 240) and soil (n = 240) were collected at random in a “W” shape across the area designated for harvesting from 24 farms, where the sheep commonly graze in October (winter) for mineral analyses. In addition, serum samples were taken via jugular vein from 20 sheep per farm from 24 farms (n = 480 samples in total) for serum minerals analyses. Mean values of macro and micro minerals were statistically compared among districts and the correlations among soil-plant-animal were statistically analyzed and correlations were regressed, as well.

**Results:**

The results revealed that there were variations for both macro and micro minerals among districts. Statistical analysis of the correlation coefficients between herbage and sheep were significantly different for most of the minerals but not for P, Cu, and Se. Many correlation regression coefficients were found significantly different among minerals of herbage, soil, and sheep serum especially those of K, Na, Fe, Mn, and Zn (between herbage and sheep serum), and Fe and Mn (between herbage and soil), Na, Fe, Mn, and Zn (between soil and sheep serum), respectively. The regression coefficient equations derived under this experiment for prediction of Ca (R^2^ = 0.618), K (R^2^ = 0.803), Mg (R^2^ = 0.767), Na (R^2^ = 0.670), Fe (R^2^ = 0.865), Zn (R^2^ = 0.950), Mn (R^2^ = 0.936), and Se (R^2^ = 0.630), resulted in significant R^2^ values.

**Conclusion:**

It is inferred that the winter herbage minerals in all the districts were below the recommended levels for macro minerals which indicated there would be some mineral deficiencies in sheep grazing the herbage in these regions. Supplemental minerals may therefore play an important role in balancing the minerals available from the herbage in winter and would lead to increased productivity in sheep on the highland areas of China. These findings could be potentially applied to the other regions for improving the livestock productivity.

## INTRODUCTION

**Figure 1:**
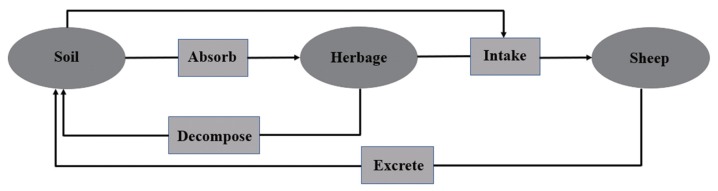
The cyclic route of mineral elements in grassland grazing system.

The Qilian Mountains, located on the northeastern margins of the Qinghai-Tibet Plateau, are composed of a string of mountains and valleys in the northwestern China. Several inland rivers including the Heihe, Shule, and Shiyang originate from the Qilian Mountains in Qinghai Province and provided valuable water resources for the neighboring lands [1]. Thus, the Qilian Mountains play a critical role in water conservation for regional sustainable development and serve as important ecological shelters in the northwest of China [2] and are also an important base of animal husbandry. Sheep grazing production is an essential component in the highlands of the northeastern part of Qilian Mountains where seasonal grazing is a predominant grazing system. The livestock graze on the natural pasture all year round, but pasture herbage cannot sufficiently provide the mineral requirements for grazing ruminants. Previous studies have reported that mineral deficiencies could have a great impact on livestock health and productivity [3,4]. Mineral deficiencies are a greater cause of losses than infectious diseases in many areas [5]. Mineral requirements of animals depend on many factors (age, stage of growth, lactation stage), and their balance with other nutrients [6]. The concentration of both macro and micro minerals in herbage can be highly variable as they are influenced by the agro-ecological factors, and the growth stage of herbage [7]. In turn, mineral availability in herbage can affect their status found in grazing animals, which may lead to mineral disorders (either excesses or deficiencies). The availability of minerals to sheep depends on many factors namely the production system or feeding practices [8]. Among many important factors, soil minerals play an important role in sheep productivity and health status because sheep obtain their required nutrients from the feeds and fodder trees, which in turn derive in nutrients from the soil. During the grazing process, livestock intentionally or unintentionally ingest a small amount of soil, so a small amount of mineral elements from soil is directly consumed by the animals (Figure 1) [9]. The contribution of soil type and its nutritive composition of herbage can greatly contribute to the performance of livestock and are dependable in each environment [10].

Assessment of minerals contained in soil and herbage where livestock graze is considered an important protocol [11]. The soil-plant-animal interrelationship has important consequences on nutritional imbalance and the productivity of livestock. The mineral profile of soil, plant, and animals has been reported by Sharma [12]; however, it has not been studied in detail in the northeastern highland of Qilian Mountains, China. Therefore, this experiment aimed to assess the status of essential minerals, both major and micro minerals, contained in soil herbage and sheep serum in areas where sheep are grazing in the highlands of Qilian Mountains to determine the mineral profile and predict mineral requirements.

## MATERIALS AND METHODS

### Animal care

The experimental procedures used in this study were approved by the Animal Ethics Committee of the Gansu Province and were performed in accordance with good scientific practices and national legislation.

### Study site and vegetation

**Figure 2:**
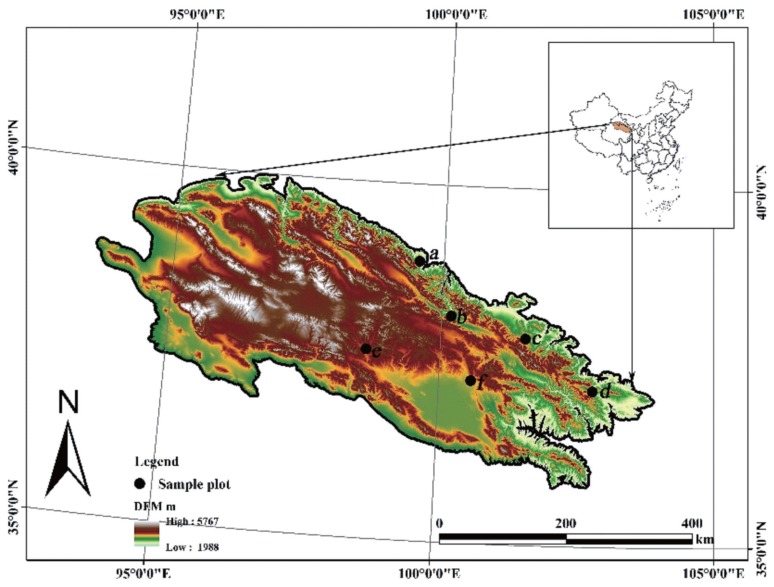
Details of the sampling sites. (a) Dahe township, (b) Qilian county, (c) Huangcheng town, (d) Tianzhu county, (e) Tianjun county, (f) Gangcha county.

Six districts in the eastern part of the Qilian Mountains of the China were the study sites (Figure 2): Dahe township (38° 54′40.09″-38°54′46.94″N, 99°31′58.61″-99°32′3.76″E, 2,877 to 3,013 m altitude); Qilian county (38°11′26.23″-38°14′43.15″N, 100°10′42.21″-100°13′21.32″E, 2,984–3,009 m altitude); Gangcha county (37°17′31.53″-37°24′38.25″N, 100°27′3.53″-100° 46′13.39″E, 3,024–3,048 m altitude); Huangcheng town (37° 53′19″-37°56′46.28″N, 101°35′29″-101°49′47.32″E, 2,498–2,880 m altitude); Tianjun county (37°40′13.39″-37°42′21.32″N, 100° 24′15.28″-100°25′18.33″E, 3,651–3,728 m altitude); Tianzhu county (36°57′49.44″-37°12′13.25″ N, 102°47′13.84″-102°59′ 54.26″E, 3,200–3,540 m altitude). The study sites were typical of the pastoral livestock production system of the highland Qilian Mountains, having short summer (July-August) and annual temperature between −0.4 and 9.6°C. In general, the vegetation in the sites consists of typical alpine meadows (Gangcha county and Tianzhu county) and grassland (Dahe township, Qilian county, Huangcheng town, and Tianjun county). Trial paddocks had been grazed at a high stocking rate prior to this study. A typical alpine meadow consists of *Stipa capillata* as dominant species, with the associated companion species being mainly *Thermopsis lanceolate*, *Kobresia myosuroides*, *Gentiana macrophylla*, *Oxytropis ochrocephala*, *Leontopodium leontopodioides*, *Potentilla chinensis*, *Poa annua*, *Kobresia myosuroides*, *Koeleria cristata*, *Plantago asiatica*, *Silene aprica*, and *Stellera chamaejasme*. A typical alpine grassland consists of *Potentilla fruticose*, *Elymus dahuricus*, and *Stipa capillata* as dominant species, with the associated companion species being mainly *Potentilla anserine*, *Iris lacteal*, *Epilobium palustre*, *Trigonotis peduncularis*, *Koeleria cristata*, *Lancea tibetica*, *Oxytropis ochrocephala*, *Poa annua*, *Leontopodium leontopodioides*, *Lomatogonium rotatum*, *Silene aprica*, *Polygonum viviparum*, *Ptilagrostis concinna*, *Deyeuxia arundinacea*, *Silene aprica*, *Potentilla chinensis*, *Dendranthema morifolium*, *Gentiana scabra*, *Medicago falcata*, *Artemisia*, *Polygonaceae*, and *Hippophae rhamnoides*.

### The management of the sheep

The Gansu Alpine Merino and Tibetan sheep were used in this experiment, with age of 7 to 10 months, and each sheep farm maintained more than 280 sheep. Eighty sheep were randomly assigned under the experimental sites. Mean body weights of the sheep were 30.7±6.4 kg. All sheep grazed on herbage and followed the local grazing management practices.

### Sampling of herbage, soil and blood of sheep

Samples of soil, herbage, and blood serum were collected from randomly selected four smallholder sheep farms of each district during October. Soil samples were taken from the soil in the layer of 0 to 15 cm depth from 10 different areas of each sheep farm, in total of 240 soil samples (40 from each district) were collected from the four districts of the Qilian Mountain. After sun-drying, the soil samples were processed through a 0.25 mm sieve for the laboratory analysis. A total of 240 herbage samples were collected at random in a “W” shape across the area designated for harvesting from the pasture where the sheep grazed in each district. The herbage samples were collected by cutting off the top portion and storing it in polythene bags for later chemical analysis. Approximately10 mL sample of blood was collected from the jugular vein of each sheep. A total of 480 blood samples were collected from sheep maintained at 24 smallholder sheep farms. Each sample of blood was then centrifuged at 2,000 g for 15 min, and the supernatant serum was then collected into polyethylene tubes and stored at −20°C until analysis.

### Mineral analysis

Approximately 0.2 g from each of the dried soil samples was digested for 20 min at 140°C and 15 atm in 5 mL of concentrated nitric acid (‘suprapur’ grade), 2 mL hydrochloric acid, 1 mL hydrofluoric acid and, 1 mL of 30% w/v hydrogen peroxide. The digested samples were cooled to room temperature, transferred to a teflon cup, 1 mL perchloric acid was added, and the hydrofluoric acid used for the analysis of total Se in the soil was removed (180°C, 10 min).

Approximately 0.2 g of each of the dried herbage samples was digested for 5 min at 140°C and 15 atm in 5 mL of concentrated nitric acid (‘suprapur’ grade) and 1 mL of 30% w/v hydrogen peroxide. The 200 μL of each serum sample was digested for 4 min at 140°C, at 14 atm in 5 mL of concentrated nitric acid (‘suprapur’ grade) in a microwave digestion system (WX-4000, Shanghai Qiyao Ltd. Co., Shanghai, China). The digested samples were cooled to room temperature, transferred to volumetric flask, and diluted to 100 mL with ‘ultrapure’ water (Sartorius Arium 611 DI; Southeast Science and Technology Co., Ltd., Guangzhou, China). The solutions were filtered (Whatman No. 1 filter paper; Guangzhou Dian Ruihua Glass Experimental Instrument Co., Ltd., Guangzhou, China) before the estimation of different minerals.

Samples (soil, herbage, and serum) were analyzed for Ca, Mg, K, Na, P, Fe, Zn, Mn, and Cu using an ICP-AES analyzer (IRIS Advantage ER/S; Lanzhou University, Lanzhou, China) [13], and the analysis of Se was carried out by atomic fluorescence spectrophotometry [14].

### Statistical analysis

The general linear model was used for analysis of variance of mineral concentrations in soil, herbage, and blood serum for different districts. Correlation coefficients of mineral content in soil, herbage, and sheep were determined from the data for mineral levels of soil, herbage, and blood serum, and the correlation between the assessed elements was estimated by Pearson’s product-moment correlation coefficient. The regression equations on the relationship among soil-plant, plant-animal, and soil-plant-animal were determined using linear regression model. All the statistical analysis was carried out using SPSS statistical analysis software (SPSS for Windows, Version 17.0, Chicago, IL, USA).

## RESULTS

### Mineral profile of soil samples

Based on the mineral analysis, there were differences between the mineral contents of soil samples collected from different districts. The mean (±standard deviation) values of Ca, K, P, Mg, Na, Fe, Mn, Zn, Cu, and Se in soils of different districts are given in Table 1. Among the macro minerals, the soil Ca content ranged from 280.9 to 552.9 mg/kg dry matter (DM), while P content was from 6.80 to 12.4 mg/kg DM. The soil minerals of the study area were classified below the recommended level for P and Mg. K and Ca contents were significantly different among districts. Among other minerals, Fe contents were relatively high (190.2±18.8 mg/kg DM against the recommended level of 2.5 mg/kg DM). Mn concentrations in soil samples in the investigated sites were significantly higher than the recommended level. Similarly, the Cu concentration was found to be higher than the recommended level in the majority of the soil samples. Evidently, all the soil samples contained higher concentrations of Fe and Zn than those of the recommended level.

### Mineral content in herbage

The concentrations of both macro and micro minerals were variable among all the districts. The data are presented in Table 2. Fe and Mn contents in herbage of different districts were invariably higher than the recommended level. Similarly, the Ca, Mg, and Se concentrations were found to be higher than the recommended level in the majority of the herbage. Except for one district, the herbage samples obtained from other five districts were deficient in K. Most of the herbage samples were deficient in P and Na. The extent of deficiency was very high in case of Na. The recommended level of Na in herbage has been reported to range between 700 and 1,000 mg/kg DM, but under the present study it was found that mean concentration of Na in herbage was only 77.14 mg/kg DM.

### Mineral contents analysis in blood serum of sheep

All mineral contents in serum of sheep are reported in Table 3. Results of blood serum of sheep analysis revealed variations in mineral contents among different districts. There were significant (p<0.05) differences in Ca, K, P, Mg, Na, Fe, Mn, Zn, and Cu concentrations in blood serum of sheep among the districts except for Se. Ca, K, Mg, Fe, Mn, and Zn concentrations in serum samples were above the maximum level of marginal range, but all the serum Na concentrations were below the marginal range (Table 3) in all the districts.

### Soil-plant-sheep interrelationship analysis

Significant correlation values were obtained between soil and herbage for Ca, P, Na, Fe, Mn, and Zn. The correlation values between herbage and sheep were significantly different for all the minerals studied except for P, Cu, and Se. Minerals (Na, Fe, Mn, and Zn) (Table 4) between soil and sheep serum were found significantly different for correlation coefficients. The correlation values between herbage and sheep were highly significant (p<0.01) for K (0.878), Na (0.749), Fe (0.825), Mn (0.951), and Zn (0.916), and between soil and herbage for Fe (0.959) and Mn (0.967). Nevertheless, such correlation coefficients were not found significantly different between those in sheep and soil except for Na (0.752), Fe (0.913), Mn (0.965), and Zn (0.935).

An assessment was conducted to assess the mineral contents in soil, herbage and sheep serum as independent values. Prediction equations that could predict the mineral contents in sheep requirements based on the mineral contents in soil and herbage are given in Table 5. Equations developed in the present study for prediction of Ca (R^2^ = 0.618), K (R^2^ = 0.803), Mg (R^2^ = 0.767), Na (R^2^ = 0.670), Fe (R^2^ = 0.865), Mn (R^2^ = 0.936), Zn (R^2^ = 0.950), and Se (R^2^ = 0.630) and were found highly significant.

## DISCUSSION

Most plants contain relatively low concentrations of Na, as compared with the requirements of animals as reported by Mcdowell [3]. But the present investigation indicated that the concentrations of Na of Qilian Mountain herbage were extremely low (Table 2) and can only meet about 8% of the sheep’s requirements (700 to 1,000 mg/kg DM) as reported by Freer [17]. However, the data obtained from sheep’s serum samples showed that the extent of deficiency of Na was not too serious, even though they were below the minimal level, as recommended by Underwood [4] (Table 3). Xin [18] concluded that the relatively sufficient Na in sheep serum could be due to soil digestion as licking soil behavior of sheep was evident. This would partly explain that although there was a low level of Na from pasture there was a relatively higher serum Na of sheep. Deficiency of Na in herbage is commonly found in the northern part of China where salt-block supplements are offered to grazing sheep to achieve improved productivity [19]. Based on the results obtained under this study, the salt-block supplement is recommended.

During early growth stage herbage usually contains a high content of P but then declines rapidly as the herbage matures [4]. Similar trend was found in the Qilian Mountain herbage, where P concentrations were below sheep requirements in most of the study areas (Table 2), which were in accordance with the findings of Masters [19]. All the serum P concentrations were within the marginal range of 31 to 46.5 mg/L [4] except in Tianjun county and Tianzhu county. This result suggested that the risk of P deficiency appears to be widespread in sheep during winter (Table 3). Those were consistent with findings reported by Long [20], in which the risk of P deficiency appears to be widespread in grazing yaks in late winter.

Previous studies showed that K concentrations in herbages would be reduced, as the herbage grows [21]. Our results showed that K concentrations in the winter herbages were lower than the recommended level of 5.0 g/kg DM [17] in most of the study areas. This result was in agreement with earlier reports Masters [19]. In the present study, although concentrations of K in soils were found to be higher than the recommended level, concentrations of the mineral in herbages obtained were lower than the recommended level. This result was similar to that given by Ashraf [22]. However, serum K concentrations were found at all above the marginal level of 93.6 to 156 mg/L [4] in all study areas. The high sheep serum of K concentration could be attributed from soil licking of sheep when grazing during the winter season.

Although, the Mg concentrations in soils were below the requirement, the concentrations of herbage minerals were relatively higher than the recommended level (Table 2). This finding is similar to that illustrated by Kumaresan [8]. Accordingly, all the serum Mg concentrations (18.6 to 28.1 mg/L) in grazing sheep were all above the limiting range of 14.6 to 18.2 mg/L [4]. Thus, the sheep has a sufficient Mg status during winter. The results agreed with the former reports that Mg deficiency in sheep seldomly occurred in the northwest of China [19].

Ca is vital to reduce the acidity of soil and is also used as a major nutrient for normal herbage growth [15]. In this study, Ca concentration in the soil was about four times higher when compared with the recommended level of 72 mg/kg DM in all testing sites. High levels of Ca contained in soil may increase Ca concentrations in herbage [23]. This is consistent with the current research results. Likewise, Mcdowell [3] reported that Ca is not likely limited in herbage diets. Our results showed that all the concentrations of herbage Ca were all within the recommended range of 1.4 to 7.0 mg/kg DM [17], furthermore, Ca in serum of sheep also was sufficient (Table 3). These results were similar to those reported earlier by Masters [19].

The possible reasons for micro mineral deficiencies across China are variable with the environment and soil structure. Sheep production is largely grass and herbage based. If the soil cannot supply sufficient trace minerals to the plants that animals are consuming, a deficiency will occur. Soil testing may indicate gross deficiencies but should only be used as a guide when considering the trace element status of livestock. The average concentrations of trace elements values of soils in China were as following [16]: Fe (2.5 mg/kg DM), Mn (5 mg/kg DM), Zn (2.5 mg/kg DM), Cu (0.3 mg/kg DM), and Se (0.5 mg/kg DM). Results of the present study revealed that the surface soils were much higher when compared with the average value of soils except for Se.

Herbage varies widely in micro mineral content due to soil type, pH, vegetation type, and horizontal distribution [24,25]. In the present experiment, it was observed that the Cu, Mn, Fe, Zn, and Se concentrations in pasture samples were higher than the recommended level [17]. The study found that Fe was excessive in herbage which may cause absorption of P, Mn, and Cu of the sheep [21]. Weak acid and neutral environmental soils are beneficial to the absorption of elemental Fe by plants [8], which may be responsible for excessive Fe content in herbage.

The abnormal content of mineral elements in animals, especially in the blood, kidney, liver and other parts, can indicate the animal has a disease or is in a poisoning state [26]. Zn and Cu are the most important essential trace minerals playing a significant role in the growth and development of animal [27]. Underwood [4] showed that serum Cu, Zn, Fe, and Se contents of serum for sheep should be from 0.19 to 0.58 mg/L, 0.4 to 0.6 mg/L, 0.19 to 2.21 mg/L, and 0.02 to 0.04 mg/L, respectively. In the ruminants, average blood Cu values of <0.5 μg/mL are a sign of severe Cu deficiency [28]. The mean concentration of Cu, observed in the present study was 0.27 mg/L, which was remarkably lower than the recommended value. These levels of serum Fe and Zn were higher than the recommended value. The results suggested that it is likely most of the sheep were deficient in Cu, however, serum Mn concentrations were in normal range. Besides, the content of elemental Se in the blood of sheep was close to the recommended value. NRC [29] pointed out that when the total Se content of the soil is less than 0.5 mg/kg DM, the lack of elemental Se can occur in livestock grazing in the area. The soil Se content in this study was found to be less than 0.5 mg/kg DM (Table 1), which further explains the lack of Se in animals of the region.

Significant correlation coefficients under this study were found for soil and herbage accounting Ca, P, Na, Fe, Mn, and Zn. The mineral contents of the herbage depend upon the type of the soil and environmental conditions in which they grow [24,25]. Usually, the content of mineral elements in the soil can meet the needs of plant growth and development. However, the effectiveness of an element is often reduced by the influence of soil properties (particle size, pH, water content, etc.), resulting in a decrease in the effective content of mineral elements in the soil [30]. As reported, there is a close relationship between soil minerals and herbage mineral contents, if the soil is low in essential minerals the uptake by roots will be impaired [31–33]. The correlation coefficients values between herbage and sheep were significantly different for all of the minerals except for P, Cu and Se. The correlation coefficient between herbage and sheep was significant for K (0.88), Na (0.75), Ca (0.74), Mg (0.67), Fe (0.83), Mn (0.95), and Zn (0.92). However, such correlations were not found between the mineral levels in sheep and in soil except for Na (0.75), Fe (0.91), Mn (0.97), and Zn (0.94). The findings under this study are opposite to those reported by Wang [11], who reported that no correlations were found between soil, herbage, and blood of sheep in Huangcheng area of Qilian Mountains. Under this current study, regression of minerals in soils and herbage revealed positive linear relationships; however, the correlation values are too small except for Ca, P, Na, Fe, Mn, and Zn. The regression equation developed to predict the mineral concentration in sheep based on the soil and herbage mineral content showed positive relationship for Ca, K, Mg, Na, Fe, Zn, Mn, and Se suggesting the possibility of prediction the mineral status in sheep.

When grazing, the mineral elements in soil and herbage will eventually be reflected in livestock [11]. The mineral elements in herbage has a crucial influence on the content and balance of mineral elements in livestock. The deficiencies of mineral elements in herbage will finally predispose to a deficient condition in serum concentrations of grazing livestock [34], and a limiting deficiency of P, Na, and K would occur under the current study but soil ingestion assuaged some of the K and Na deficiency. In addition, livestock of different species and physiological periods may require different levels of minerals. For instances, grazing cows require higher Mg levels during lactation [35]. Growing young animals and productive animals would require higher mineral levels than other physiological stages [36]. Although the results obtained under this trial were from castrated sheep, the findings could be further implemented to other livestock together with long term feeding trials to offer more information on other species of livestock.

The experiment carried out in the high land of Qilian Mountain, northwestern of China, assessing the mineral contents in soil, herbage and sheep serum, resulted in useful information on the mineral status and the soil-herbage-sheep relationship. The results revealed that there were variations of both macro and micro minerals among districts. Among others, Na and P deficiency could be a prevalent deficiency in sheep. Hence, salt-block containing these minerals should be supplemented to ensure better productivity of sheep grazing in the highlands of China.

## Figures and Tables

**Table 1:** Concentrations (mg/kg dry matter) of mineral elements in soil (mean±SD)

District name	Ca	K	P	Mg	Na	Fe	Mn	Zn	Cu	Se1)
Dahe township	465.78±16.54^c^	153.93±8.75^d^	8.60±0.27^d^	17.41±0.19^c^	13.92±0.33^b^	144.69±7.92^e^	6.88±0.48^e^	113.65±4.53^d^	0.29±0.01^c^	0.09±0.01^d^
Tianjun county	393.35±15.31^d^	147.73±12.43^e^	12.49±1.18^a^	10.74±0.53^f^	15.35±0.49^a^	297.47±12.36^a^	18.62±1.21^a^	116.75±9.46^ab^	0.46±0.03^ab^	0.13±0.00^b^
Qilian county	485.36±17.07^b^	142.73±12.43^f^	8.71±1.39^d^	15.68±0.35^d^	14.07±1.70^b^	153.85±9.80^d^	8.57±0.18^d^	115.82±4.45^c^	0.45±0.05^ab^	0.11±0.01^c^
Tianzhu county	280.97±13.27^f^	212.48±12.42^a^	9.47±0.41^c^	18.03±1.37^b^	14.13±0.83^b^	208.21±14.47^b^	11.06±0.50^b^	116.14±3.82^bc^	0.48±0.02^a^	0.08±0.01^e^
Gangcha county	296.91±12.04^e^	159.64±13.81^c^	6.80±1.22^e^	14.91±0.38^e^	13.47±0.30^b^	154.91±11.42^d^	8.89±0.34^d^	115.72±6.19^c^	0.43±0.02^b^	0.10±0.01^d^
Huangcheng town	552.96±22.38^a^	169.98±11.09^b^	11.56±2.38^b^	20.92±2.47^a^	15.81±1.81^a^	182.32±11.27^c^	10.33±0.50^c^	117.15±2.97^a^	0.44±0.02^b^	0.18±0.01^a^
Significance of region	<0.0001	<0.0001	<0.0001	<0.0001	<0.0001	<.0001	<0.0001	<0.0001	<0.0001	<0.0001
Overall (mean±SD)	412.56±16.76	164.42±13.93	9.60±1.95	16.28±3.19	14.46±0.97	190.24±18.84	10.73±1.62	115.87±3.89	0.44±0.05	0.11±0.03
Recommended level2)	72	37	17	30	-	2.5	5	2.5	0.3	0.5

SD, standard deviation.

1) Total concentration for Se in soils.

2) Recommended levels for Ca, Mn, Zn, and Cu from Rhue [15]; Recommended levels for P, Mg, K, Fe, Co, and Se from Viets [16].

a–f Means with different superscripts between districts differ significantly (p<0.01).

**Table 2:** Macro and micro mineral concentrations (DM) in herbage samples (mean±SD)

District name	Ca	K	P	Mg	Na	Fe	Mn	Zn	Cu	Se
	
	------------------------------------ g/kg -----------------------------------				--------------------------------------------------------- mg/kg -----------------------------------------------------					
Dahe twnship	8.83±0.23^b^	2.85±0.19^c^	0.76±0.02^c^	1.26±0.03^e^	51.51±3.08^cd^	227.04±12.98^e^	62.31±3.70^f^	19.20±1.48^d^	13.20±2.32^c^	0.04±0.00^c^
Tianjun county	9.82±0.09^a^	3.58±0.08^b^	0.96±0.01^a^	1.72±0.02^a^	68.17±2.64^b^	334.73±14.27^a^	93.92±2.69^a^	32.25±0.73^a^	15.88±2.67^a^	0.07±0.00^a^
Qilian county	8.70±0.13^b^	2.67±0.17^c^	0.78±0.03^c^	1.65±0.02^b^	55.33±2.42^c^	237.21±13.27^d^	65.46±0.81^e^	21.19±0.85^c^	15.26±0.79^ab^	0.03±0.00^d^
Tianzhu county	5.92±0.14^d^	3.48±0.10^b^	0.66±0.03^d^	1.56±0.03^d^	69.01±9.90^b^	285.57±11.40^b^	77.78±4.83^b^	26.82±0.69^b^	13.29±1.70^c^	0.05±0.00^b^
Gangcha county	7.99±0.11^c^	5.60±0.29^a^	0.68±0.02^d^	1.18±0.02^f^	45.17±2.86^d^	264.86±12.18^c^	73.42±1.23^d^	21.47±1.62^c^	13.41±1.55^c^	0.05±0.01^b^
Huangcheng town	8.77±0.20^b^	2.70±0.20^c^	0.85±0.02^b^	1.60±0.04^c^	173.67±8.82^a^	265.59±11.78^c^	75.58±2.58^c^	21.61±1.55^c^	14.55±0.47^b^	0.07±0.00^a^
Significance of region	<0.0001	<0.0001	<0.0001	<0.0001	<0.0001	<0.0001	<0.0001	<0.0001	<0.0001	<0.0001
Overall (mean±SD)	8.34±1.27	3.48±0.17	0.78±0.11	1.49±0.21	77.14±7.49	269.17±15.95	74.75±8.34	23.76±4.55	14.26±1.19	0.05±0.01
Recommended level1)	1.4–7.0	5.0	0.9–3.0	0.9–1.2	700–1000	40	20–25	9–20	4–14	0.05

DM, dry matter; SD, standard deviation.

1) Recommended level according to nutrient requirements of sheep [17]. When a range is given, the higher values are for rapidly growing, pregnant, or lactating sheep and the lower values are for those at maintenance or with a low level of production.

a–f Means with different superscripts between districts differ significantly (p<0.01).

**Table 3:** Macro and micro mineral concentrations (mg/L) in blood serum of sheep (mean±SD)

District name	Ca	K	P	Mg	Na	Fe	Mn	Zn	Cu	Se
Dahe township	109.61±2.47^d^	183.33±12.07^e^	40.12±1.48^d^	21.08±1.30^d^	2961.00±87.24^d^	6.62±1.31^c^	0.13±0.00^c^	1.21±0.05^b^	0.17±0.02^c^	0.02±0.00
Tianjun county	112.46±1.72^c^	202.01±12.62^d^	49.02±1.04^a^	24.45±1.21^b^	3,165.00±121.42^b^	7.73±0.43^a^	0.15±0.02^a^	1.39±0.06^a^	0.45±0.04^a^	0.03±0.01
Qilian county	119.39±2.27^b^	171.57±12.70^f^	43.22±1.25^c^	23.07±3.94^c^	3,052.17±154.81^c^	6.81±0.38^bc^	0.13±0.01^bc^	1.28±0.04^b^	0.19±0.01^c^	0.02±0.00
Tianzhu county	111.92±2.18^cd^	251.28±11.89^a^	49.26±2.18^a^	24.15±3.27^bc^	3,145.83±64.84^b^	7.29±0.27^ab^	0.14±0.01^b^	1.38±0.07^a^	0.29±0.03^b^	0.02±0.00
Gangcha county	98.47±2.01^e^	213.81±11.99^c^	45.39±0.62^b^	18.63±2.91^e^	2,867.33±87.39^e^	6.97±0.41^bc^	0.13±0.01^c^	1.28±0.06^b^	0.23±0.04^c^	0.03±0.00
Huangcheng town	123.24±2.16^a^	231.77±11.92^b^	43.59±1.50^c^	28.12±2.31^a^	3,273.50±83.06^a^	7.14±0.41^b^	0.13±0.00^c^	1.23±0.07^b^	0.31±0.02^b^	0.03±0.00
Significance of region	<0.0001	<0.0001	<0.0001	<0.0001	<0.0001	<0.0001	<0.0001	<0.0001	<0.0001	>0.2781
Overall (mean±SD)	112.52±8.19	208.96±17.65	45.10±3.55	23.25±4.15	3,077.47±140.62	7.09±0.51	0.13±0.01	1.29±0.09	0.27±0.07	0.03±0.01
Recommended level 1)	70–80	93.6–156	31–46.5	14.6–18.2	3,320–3,335	0.19–2.21	0.002	0.4–0.6	0.19–0.58	0.02–0.04

SD, standard deviation.

1) Recommended levels for Ca, K, P, Mg, Na, Fe, Mn, Zn, Cu, Co, and Se from Underwood [4].

a–f Means with different superscripts between districts differ significantly (p<0.01).

**Table 4:** Soil-plant-animal relationship (correlation) in respect to macro and micro mineral status

Mineral	Ca	K	P	Mg	Na	Fe	Mn	Zn	Cu	Se
Soil-plant										
Pearson correlation value	0.592*	0.059	0.762*	−0.143	0.683*	0.959**	0.967**	0.805*	0.124	−0.528
p-value	0.044	0.730	0.040	0.407	0.042	0.008	0.007	0.023	0.624	0.324
Plant-sheep										
Pearson correlation value	0.741*	0.878**	−0.228	0.672*	0.749**	0.825**	0.951**	0.916**	0.124	0.786
p-value	0.017	0.007	0.264	0.026	0.008	0.004	0.001	0.002	0.624	0.079
Soil–sheep										
Pearson correlation value	0.227	0.232	−0.140	0.731	0.752**	0.913**	0.965**	0.935**	0.433	−0.319
p-value	0.182	0.173	0.495	0.058	0.008	0.006	0.002	0.004	0.073	0.497

* Significant at 0.05 level,

** significant at 0.01 level.

**Table 5:** Regression equation on soil-plant-animal continuum in relation to mineral status

Mineral	Regression equation to predict mineral content in pasture based on the mineral status of soil	R^2^	Regression equation to predict mineral content in sheep based on the mineral status of pasture	R^2^	Regression equation to predict mineral content in sheep based on the mineral status of soil	R^2^	Regression equation to predict mineral content in sheep based on the mineral status of soil and plant	R^2^
Ca	A = 0.008B+5.241	0.650	C = 1.460A+100.342	0.549	C = 0.060B+87.621	0.052	C = 0.076B–2.087A+98.559	0.618
K	A = 0.003B+3.046	0.004	C = 6.115A+187.672	0.771	C = 1.030B+39.589	0.054	C = 1.018B+4.756A+25.101	0.803
P	A = 0.033B+0.464	0.581	C = −6.817A+49.508	0.052	C = −0.479B+48.620	0.020	C = −0.948B+22.597A+37.577	0.263
Mg	A = −0.009B+1.648	0.020	C = 11.109A+6.616	0.731	C = 0.367B+17.267	0.138	C = 0.480B+12.163A–2.778	0.767
Na	A = 31.575B–379.525	0.563	C = 2.352A+2,896.054	0.562	C = 108.414B+1,509.506	0.752	C = 63.969B+1.408A+2,043.723	0.670
Fe	A = 0.655B+144.384	0.920	C = 0.011A+3.903	0.681	C = 0.007B+5.600	0.833	C = −0.005B+0.019A+2.906	0.865
Mn	A = 2.594B+ 47.068	0.934	C = 0.001A+0.079	0.904	C = 0.002B+0.111	0.931	C = 0.001B+0.001A+0.087	0.936
Zn	A = 3.925B–430.092	0.648	C = 0.015A+0.887	0.838	C = 0.073B–7.219	0.874	C = 0.037B+0.009A–3.251	0.950
Cu	A = 3.162B+12.693	0.015	C = 0.035A–0.229	0.158	C = 0.824B–0.085	0.187	C = 0.724B+0.032A–0.485	0.308
Se	A = −0.675B+0.124	0.278	C = −0.099A+0.028	0.418	C = 0.312B–0.012	0.102	C = 0.340B+0.041A–0.018	0.630

A mineral content in herbage; B mineral content in soil; C mineral content in sheep.
